# Symptom aggravation after withdrawal of metal chelating agent therapy in patients with Wilson's disease

**DOI:** 10.1002/brb3.3170

**Published:** 2023-07-26

**Authors:** Xiangxue Zhou, Jian Liao, Yinjie Liu, Haolin Qin, Xia Xiao

**Affiliations:** ^1^ Department of Neurology The First Affiliated Hospital, Sun Yat‐sen University, Guangdong Provincial Key Laboratory of Diagnosis and Treatment of Major Neurological Diseases, National Key Clinical Department and Key Discipline of Neurology Guangzhou China; ^2^ Department of Imaging The First Affiliated Hospital of Sun Yat‐sen University Guangzhou China

**Keywords:** disease stage, drug discontinuance, metal chelating agent, symptoms aggravated, Wilson disease

## Abstract

**Objective:**

To study the aggravation of clinical symptoms after discontinuation of metal chelating agent therapy in Wilson's disease (WD) patients, analyze the causes of aggravation, and observe the prognosis.

**Methods:**

40 WD patients (cerebral type 30 cases and hepatic type 10 cases) who stopped using metal chelating agent were selected, 40 WD patients with normal therapy, and 10 normal control cases were selected. All patients underwent neurological symptom evaluation using modified Young scale, Child‐Pugh liver function grading, metal metabolism, and disease typing. Magnetic sensitivity imaging (SWI), diffusion tensor imaging (DTI), and magnetic resonance spectroscopy imaging (MRS) were performed. According to the imaging results, WD patients were divided into metal deposition stage, fiber damage stage, and neuron necrosis stage. All patients were treated with metal chelating agent for 6 months.

**Results:**

The score of modified Young scale in drug withdrawal group was lower than that in normal treatment group before drug withdrawal (*p* = .032). The score of modified Young scale was higher after drug withdrawal than before (*p* = .011). The number of Child‐Pugh B‐grade patients after drug withdrawal was more than that before drug withdrawal and in normal treatment group. The proportion of patients in the stage of neuronal necrosis after drug withdrawal (25%) was higher than that before drug withdrawal (10%) (*p* = .025). After drug withdrawal, urine copper was significantly higher than that before drug withdrawal and in the normal treatment group (*p* = .032, .039). After the withdrawal group resumed metal chelating agent treatment, 34.2% of neurological symptoms worsened.

**Conclusions:**

WD patients showed neurological symptoms aggravation and increased liver injury after metal chelating agent withdrawal. Increased metal deposition and new nerve injury occurred in the brain. After re‐treatment, the aggravated neurological symptoms of WD patients are difficult to reverse.

## Graphical abstract

WD patients showed neurological symptoms aggravation and increased liver injury after metal chelating agent withdrawal. Increased metal deposition and new nerve injury occurred in the brain. After re‐treatment, the aggravated neurological symptoms are difficult to reverse.

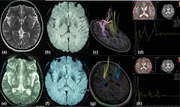

## 1 INTRODUCTION

Wilson disease (WD) is an autosomal recessive disorder of copper metabolism. It is characterized by insufficient ceruloplasmin synthesis and copper disturbance in biliary discharge (Liang, 2001), with cirrhosis, extrapyramidal symptoms, and corneal K–F ring as the main manifestations. According to the clinical symptoms of patients, WD patients can be divided into hepatic type (mainly cirrhosis symptoms, no obvious neurological, and psychiatric symptoms), cerebral type (mainly neurological and/or psychiatric symptoms and mild liver symptoms), and mixed type. Copper excretion is the main treatment method. Currently, metal chelating agents commonly used to drive copper include penicillamine (DPA), sodium dimercaptopropanesulfonate (DMPS), dimercaptosuccinic acid (DMSA), and so on (Aggarwal & Bhatt, 2018, 2020; Fernando et al., 2020; Mulligan & Bronstein, 2020). However, the metal chelating agent has some side effects, such as worsening neurological symptoms, drug‐induced liver damage, gastrointestinal symptoms, and rash. There is a high proportion of treatment interruption in WD patients. The withdrawal rate of metal chelating agent is 20%–30% (Zhou et al., 2020). We found that a high proportion of clinical symptoms worsened in WD patients after the withdrawal of metal chelating agent. In addition, the response to metal chelating agent therapy was poor. At present, there are few studies on the aggravation of clinical symptoms and prognosis of WD patients after metal chelating agent withdrawal. In this paper, the reasons for the withdrawal of metal chelating agent in WD patients, the progress of clinical symptoms after drug withdrawal, the causes of symptom aggravation, and the response to treatment were studied.

## 2 MATERIALS AND METHODS

### 2.1 Participants

A total of 40 WD patients (cerebral type 30 cases and hepatic type 10 cases) with metal chelator interruption in the First Affiliated Hospital of Sun Yat‐sen University, China from July 2007 to March 2022 were collected. A total of 40 WD patients (cerebral type 30 cases and hepatic type 10 cases) with normal treatment matched with age and disease course were selected. All the selected cases met the diagnostic criteria for WD (Liang, 2001): ceruloplasmin <0.2 g/L, 24 h urine copper >100 μg, corneal K–F ring positive, family history of positive.

10 healthy volunteers matched with the age of the patients were selected.

All subjects signed informed consent, which was approved by the Hospital Ethics Committee.

### 2.2 Methods

#### 2.2.1 Clinical data collection

All WD patients and normal controls were tested for urinary copper, serum copper, and liver function. The liver function was graded for Child‐Pugh scoring (Table 1). The metal index was determined by atomic absorption spectrophotometer flame method. The measuring instrument was Hitachi Z‐5000 atomic absorption spectrophotometer. Hollow CATHODE lamp: Copper L233 wavelength: 324.9 nm, current: 10 mA.

**TABLE 1:** Child‐Pugh scoring.

	1 point	2 point	3 point
Hepatic encephalopathy (grade)	No	1–2 grade	3–4 grade
Ascites	No	Mild	moderate‐severe
Total bilirubin (μmol/L)	<34	34–51	>51
Albumin (g/L)	>35	28–35	<28
PT extension (s)	<4	4–6	>6

*Note*: Classification: grade A: 5–6 points; grade B: 7–9 points; grade C: 10–15 points.

The modified Young scale (Zhou et al., 2011) (including eight categories of language, throat muscle tone, limb muscle tone, ataxia, tremor, dance‐like movement, gait, and advanced neural activity) was used to score WD patients for neurological symptoms. According to the clinical symptoms of cerebral type WD patients and the scores of modified Young scale, cerebral type WD patients can be divided into the following four types: (1) involuntary movements; (2) Parkinson's symptoms; (3) oral‐mandibular dystonia; (4) mental disorders.

Neurological symptom score, metal metabolism, liver function, and imaging data of WD patients were collected. The use of metal chelating agent in WD patients in drug withdrawal group and the reasons for stopping metal chelating agent were collected.

#### 2.2.2 Imaging examination and disease staging

Magnetic sensitive imaging (SWI) was performed on WD patients and normal controls 3.0T Philips Achieva Nova Dual Plus Superconducting MR scanner and 8‐channel SENSE Head coil. The scanning sequence is 3D‐FFE sequence, TR/TE: 60 ms/40 ms, NSA: 1, FOV: 240 × 240 mm^2^, fractional anisotropy (FA): 18, resolution: 1 mm × 1 mm × 0.5 mm, echo shift: ON. SPIN software was used to perform high‐pass filtering on the original phase image, and a 64 × 64 low‐pass filter was used to divide the original image by the filtered *K*‐space data to obtain the corrected phase diagram. Five regions of interest (ROIs) were selected: globus pallidus (GP), caudate nucleus (CA), putamen (PU), thalamus (TH), and substantia nigra (SN). The ROI was set up by two experienced neuroimaging physicians. The corrected phase (CP) values of each ROI were measured.

Diffusion tensor imaging (DTI) examination was performed on WD patients and normal controls. FA of each ROI was measured.

Magnetic resonance spectroscopy imaging (MRS) monomer signals of patients were collected, MRS signals of each ROI were collected, and magnetic resonance signals of *N*‐acetylaspartate (NAA), Cholitle (Cho), creatine (Cr), and other mixtures were transformed by Fourier transform or two‐dimensional spectrum graph to calculate the area under the peak. Three main peaks of NAA, Cr, and Cho were located at the displacement of 2.03, 3.0, and 3.2 PPM, respectively. The subpeak area integral is the relative concentration of the substance in the ROI, and the NAA/Cr ratio is calculated.

According to the imaging results of DTI, SWI and MRS, WD patients were divided into metal deposition stage (SWI imaging showed obvious abnormal CP value, DTI imaging showed no obvious abnormal FA value, and MRS imaging showed no obvious NAA peak drop), fiber damage stage (DTI imaging showed obvious abnormal FA value and MRS imaging showed no obvious NAA peak decline), and neuron necrosis stage (MRS imaging showed obvious NAA peak decline).

#### 2.2.3 Observation of therapeutic effect

All patients were treated with metal chelating agent for 6 months. DPA, DMPS, or DMSA were selected for treatment according to the clinical type of patients (Zhou et al., 2020; Zhou et al., 2019) (selection method: DPA was selected for WD patients with hepatic type, cerebral type 1 and cerebral type 4. DMPS and DMSA were used to treat cerebral type 2 and 3 WD patients). DPA treatment: 500–750 mg/day, three times a day. The dosage of DMPS was 5 mg/kg. DMPS was dissolved in 500 mL normal saline and intravenous infusion, once a day for 14 consecutive days. After discharge, DMSA therapy was given. The treatment of DMSA was 10 mg/kg twice a day. Patients intolerant to DPA, DMPS, and DMSA were treated with zinc gluconate alone. Neurological symptom scores, liver function, copper metabolism, and imaging examination were evaluated after treatment.

### 2.3 Statistical analysis

The results were recorded by *x* ± *s*. *t*‐Test and analysis of variance were used to compare the clinical indicators of WD patients in the drug withdrawal group and the normal treatment group before and after treatment. SPSS13.0 software was used for statistical analysis.

## 3 RESULTS

### 3.1 Basic information of WD patients and normal controls (Table 2)

There were no significant differences in age, sex, and course of disease between the drug withdrawal group and the normal treatment group.

### 3.2 Drug withdrawal status of drug withdrawal group

Classification of WD patients before drug withdrawal in the drug withdrawal group was as follow: 15 cases of type 1, 8 cases of type 2, 5 cases of type 3, 2 cases of type 4, and 10 cases of hepatic type. The classification of WD patients after drug withdrawal in the drug withdrawal group was as follows: 5 cases of type 1, 16 cases of type 2, 13 cases of type 3, 1 case of type 4, and 5 cases of hepatic type. Normal treatment components: 5 cases of type 1, 13 cases of type 2, 10 cases of type 3, 2 cases of type 4, and 10 cases of hepatic type.

In the withdrawal group, metal chelating agent was used before drug withdrawal: DPA (25 cases), DMPS + DMSA (12 cases), and zinc gluconate alone (3 cases). Metal chelating agent used in the normal treatment group was as follow: DPA (17 cases), DMPS + DMSA (22 cases), and zinc gluconate alone (1 case).

The reasons for drug withdrawal in the drug withdrawal group were side effects (13 cases), symptom improvement (22 cases), and economic reasons (5 cases).

Duration of drug withdrawal was as follow: less than 6 months (17 cases), 6 months to 1 year (13 cases), 1–2 years (7 cases), and 2–3 years (3 cases).

The reasons for recovery of treatment were as follows: neurological symptoms were aggravated (21 cases), hepatic type patients had neurological symptoms (5 cases), liver function was aggravated (6 cases), and family members required treatment (8 cases).

The score of modified Young scale before drug withdrawal in drug withdrawal group was lower than that in normal treatment group (*p* = .032). The score of modified Young scale was higher after drug withdrawal than before in drug withdrawal group (*p* = .011).

The number of Child‐Pugh B‐grade patients after drug withdrawal was more than that before drug withdrawal and in normal treatment group (Table 2). In the drug withdrawal group, liver enzymes were significantly increased in six patients, bilirubin was significantly increased in five patients, coagulation time was prolonged in five patients, albumin was decreased in three patients, and ascites was observed in one patient after withdrawal.

**TABLE 2:** Comparison of basic conditions of Wilson's disease (WD) patients and normal controls.

	Drug withdrawal group before drug withdrawal	Drug withdrawal group after drug withdrawal	Normal treatment group	Normal control
Number	40	40	40	10
Gender (male/female)	24/16	24/16	20/20	5/5
Age (year)	22 ± 7	23 ± 8	20 ± 6	20 ± 3
Cerebral/hepatic/mixed type	30/10/0	33/5/2	30/10/0	
Course of disease (month)	24 ± 11	34 ± 15	26 ± 8	0
Modified Young scale	11.05 ± 3.26^a^	20.31 ± 7.35^a^, ^b^	17.52 ± 7.20^a^	0
Child Pugh grade (A/B)	38/2	35/5	38/2	
Total bilirubin (μmol/L)	38.05 ± 7.33^a^	40.46 ± 9.57^a^	36.22 ± 5.28^a^	20.33 ± 4.50
Albumin (g/L)	33.05 ± 5.42	30.73 ± 7.35	32.51 ± 5.07	37.26 ± 3.14
PT extension (s)	5.07 ± 2.15^a^	5.25 ± 2.40^a^	4.79 ± 1.22^a^	3.87 ± 1.55
Serum copper (mg/L)	0.28 ± 0.08^a^	0.35 ± 0.09^a^	0.29 ± 0.07^a^	0.81 ± 0.10
Urine copper (μg/day)	280 ± 119^a^	625 ± 372^a^, ^b^	328 ± 123^a^	32 ± 8

^a^ Compared with normal treatment group, *p* ≤ .05.

^b^ Compared with before drug withdrawal, *p* ≤ .05.

**TABLE 3:** Imaging staging of Wilson's disease (WD) patients.

		Drug withdrawal group before drug withdrawal	Drug withdrawal group after drug withdrawal	Normal treatment group	Control
Number (cases)		40	40	40	10
Metal deposition stage		10	5	11	
Fiber damage stage		26	25	21	
Neuronal necrosis stage		4	10	8	
SWI CP values	PU	2190 ± 154	1902 ± 326	2053 ± 227	2265 ± 102
	GP	1775 ± 262	1430 ± 84^a^, ^b^	1622 ± 231^b^	2094 ± 117
	TH	2039 ± 144	2112 ± 302	2164 ± 379	2352 ± 52
	SN	1627 ± 305^b^	1303 ± 93^a^, ^b^	1516 ± 164^b^	2067 ± 74
	CA	2248 ± 153	2105 ± 236	2052 ± 198	2296 ± 75
DTI FA values	PU	0.26 ± 0.10	0.18 ± 0.07^a^, ^b^	0.24 ± 0.08	0.28 ± 0.03
	GP	0.25 ± 0.07	0.20 ± 0.07^b^	0.21 ± 0.09^b^	0.29 ± 0.06
	TH	0.37 ± 0.03	0.35 ± 0.12	0.33 ± 0.08	0.40 ± 0.02
	SN	0.50 ± 0.11	0.45 ± 0.09^b^	0.47 ± 0.13	0.55 ± 0.05
	CA	0.30 ± 0.07	0.32 ± 0.09	0.34 ± 0.09	0.36 ± 0.07
MRS NAA/Cr	PU	3.47 ± 0.65	1.82 ± 0.73^a^, ^b^	2.63 ± 1.03^b^	3.83 ± 0.70
	GP	3.35 ± 1.03	1.92 ± 0.46^a^, ^b^	2.75 ± 1.20^b^	3.95 ± 0.56
	TH	4.45 ± 1.21	4.52 ± 1.03	4.72 ± 1.76	5.25 ± 0.12
	SN	1.64 ± 0.57	1.58 ± 0.39	2.03 ± 1.01	1.30 ± 0.08
	CA	4.49 ± 1.52	4.37 ± 1.23	4.65 ± 1.18	5.19 ± 0.12

Abbreviations: CA, head of caudate nucleus; FA, fractional anisotropy; GP, globus pallidus; MRS, magnetic resonance spectroscopy; PU, putamen; SN, substantia nigra; TH, thalamus.

^a^ Statistically significant compared to that before drug withdrawal (*p* ≤ .05).

^b^ Statistically significant compared to the normal control (*p* ≤ .05).

**TABLE 4:** The modified Young scale scores in cerebral type Wilson's disease (WD) patients before and after resumed metal chelating agent therapy.

Drug		Drug withdrawal group	Normal treatment group
DPA	Number (cases)	7	7
	Pretreatment score	13.15 ± 9.32	11.27 ± 9.05
	Posttreatment score	17.21 ± 7.32^a^, ^b^	9.25 ± 5.31
DMPS + DMSA	Number (cases)	26	22
	Pretreatment score	22.16 ± 5.22	19.42 ± 9.25
	Posttreatment score	21.72 ± 8.07	17.07 ± 10.27
Zn	Number (cases)	2	1
	Pretreatment score	23.20 ± 5.26	20.41 ± 4.23
	Posttreatment score	22.33 ± 7.21^b^	17.05 ± 8.16

Abbreviations: DMPS, dimercaptopropanesulfonate; DMSA, dimercaptosuccinic acid.

^a^ Compared with normal treatment group, *p* ≤ .05.

^b^ Compared with before treatment, *p* ≤ .05.

After drug withdrawal, the urine copper was significantly higher than that before drug withdrawal and in the normal treatment group (*p* = .032, .039).

### 3.3 Imaging stage of WD patients (Table 3, Figure 1)

According to DTI, SWI, and MRS imaging results, patients were divided into metal deposition stage, fiber damage stage, and neuron necrosis stage. The proportion of patients in the stage of neuron necrosis after drug withdrawal (25%) was significantly higher than that before drug withdrawal (10%) (*p* = .025). The proportion of metal deposition stage in normal treatment group (27.5%) was higher than that in drug withdraw group (12.5%) (*p* = .022).

**FIGURE 1:**
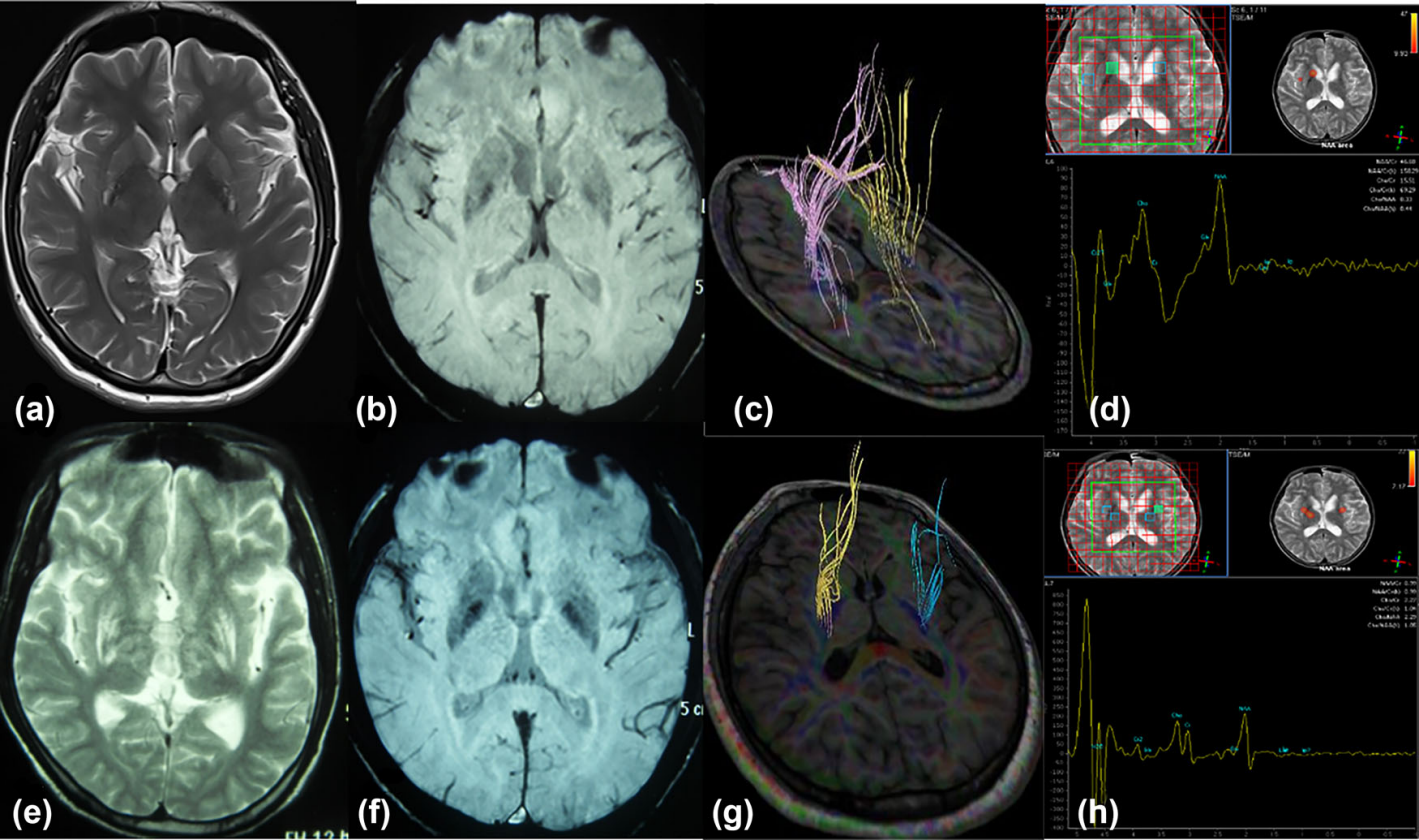
Imagings of a 16‐year‐old male patient with cerebral type Wilson's disease (WD) before and after drug withdrawal. Before drug withdrawal, the patient presented with slurred speech and limb tremor, and the modified Young scale score was 9 points. MRIT2 imaging of the head showed slightly hyperintensity in bilateral putamen and hypointensity in globus pallidus (a). SWI imaging showed hypointensity in putamen and globus pallidus (b). DTI fiber reconstruction showed that the number of putamen associated fibers was in the normal range (c). Magnetic resonance spectroscopy (MRS) imaging showed that NAA/Cr in putamen was in normal range (d). According to the imaging results, the patient was in the metal deposition stage. Eight months after drug withdrawal, the patient developed aggravated slurred speech, and increased limb muscle tone and abnormal gait. The modified Young scale score was 24 points. Head MRIT2 imaging showed that the range and degree of hyperintensity in bilateral putamen and globus pallidus were worse than those before drug withdrawal (e). SWI imaging showed that the putamen and globus pallidus were significantly lower than those before drug withdrawal (f). DTI fiber reconstruction showed a decrease in the number of putamen associated fibers (g). MRS imaging showed that NAA/Cr of putamen decreased significantly (h). According to the imaging results, the patient was in the neuronal necrosis stage.

In the drug withdrawal group, CP values of GP (*p* = .039) and SN (*p* = .026) on SWI imaging were lower than before drug withdrawal. On DTI imaging, (*p* = .016) FA value of PU was lower than before drug withdrawal. On MRS imaging, NAA/Cr of PU and GP was lower than before drug withdrawal (*p* = .033, .042). CP value of GP on SWI imaging in the drug withdrawal group was lower than that in the normal treatment group (*p* = .028).

### 3.4 Effect of resumed metal chelating agent therapy (Table 4)

All patients in the drug withdrawal group resumed metal chelating agent therapy. Twelve patients (5 hepatic) were treated with DPA, 26 with DMPS + DMSA, and 2 with zinc gluconate alone. In the normal treatment group, the original treatment regimen was maintained: DPA (17 cases, 10 hepatic type), DMPS + DMSA (22 cases), and zinc gluconate alone (1 case).

In the withdrawal group, neurological symptoms improved in 5 patients (14.3%), not improved in 18 patients (51.4%), and aggravated in 12 patients (34.2%) after 6 months of metal chelating agent treatment. In the normal treatment group, neurological symptoms improved in 14 patients (46.7%), not improved in 8 patients (26.7%), and aggravated in 8 patients (26.7%).

The modified Young scale of WD patients in the drug withdrawal group after DPA treatment was higher than that before treatment (*p* = .036), and higher than that in the normal treatment group (*p* = .029).

## 4 DISCUSSION

WD is a disorder of copper metabolism. Abnormal deposits of metals such as copper and iron lead to neurological and liver symptoms. According to the clinical characteristics of WD patients, WD patients can be divided into hepatic type, cerebral type, and mixed type. According to the characteristics of neurological symptoms, WD patients with cerebral type can be divided into involuntary movements, Parkinson's symptoms, oromandibular dystonia, and mental disorders (Zhou et al., 2019, 2020). There are great differences in drug selection and drug response among WD patients with different types. At present, metal chelating agent copper excretion is an important treatment for WD (Li et al., 2022). Commonly used metal chelating agents include DPA, DMPS, DMSA, and so on. However, we found that discontinuation of metal chelators was frequent. Copper excretion with metal chelators can lead to aggravation of neurological symptoms, and the proportion and degree of aggravation of neurological symptoms in WD patients caused by different metal chelators are different (Aggarwal & Bhatt, 2018, 2020; Fernando et al., 2020; Mulligan & Bronstein, 2020; Zhou et al., 2019, 2020). Metal chelating agent has many other side effects, such as liver damage, gastrointestinal reaction, and myelosuppression. In the process of copper excretion with metal chelating agent, some WD patients stopped taking drugs because they could not tolerate the aggravation of neurological symptoms or side effects of drugs. In addition, some WD patients stopped treatment privately due to economic factors and personal adherence problems. We found that after drug withdrawal, these WD patients experienced a rapid deterioration of clinical symptoms. However, there is no systematic study on the reasons for the withdrawal of metal chelating agent, the aggravation of clinical symptoms, and the prognosis of these WD patients. In this study, factors, disease progression, and prognosis of metal chelating agent withdrawal in WD patients were analyzed.

We found that WD patients who stopped metal chelating agent had certain characteristics in clinical symptom classification. The patients in drug withdrawal group were mainly type of involuntary movement, who took tremor as main symptom. In the normal treatment group, increased muscle tension was the main symptom. Tremor has little effect on patients and is not easy to cause their attention. Meanwhile, the neurological symptom scores before drug withdrawal in the drug withdrawal group were lower than that in the normal treatment group. Mild neurological symptoms may be one of the reasons why patients ignore their condition and stop treatment. Since most of the patients in the drug withdrawal group had involuntary movement, these patients responded well to metal chelating agent therapy. The improvement in neurological symptoms also leads patients to stop treatment in the mistaken belief that the disease is cured. In addition, the proportion of WD patients treated with DPA in the drug withdrawal group was higher than that in the normal treatment group (25:17). Previous studies had found that DPA has a higher incidence of side effects than DMPS and DMSA (Zhou et al., 2020). In this study, 13 out of 40 patients in the discontinuation group stopped treatment because of side effects. Therefore, we analyzed that the reasons for WD patients to stop metal chelating agent therapy were clinical type of patients, good treatment effect, and drug side effects.

One of the reasons for WD patients who stopped treatment to see a doctor again was the aggravation of clinical symptoms. Among the enrolled WD patients who stopped treatment, 32/40 showed worsening clinical symptoms. Aggravation of neurological symptoms is most likely to occur. The neurological symptom scores of patients in the drug withdrawal group were significantly higher than those before discontinuation. Most patients had slurred speech, and increased muscle tension. Cerebral type WD patients in the drug withdrawal group showed changes in clinical classification after drug withdrawal. The proportion of patients with increased limb muscle tone (type 2) and oral and mandibular muscle tone (type 3) was significantly higher after drug withdrawal. In addition, some patients with hepatic type WD showed neurological symptoms after drug withdrawal, mainly including tremor and dystonia. In addition, some patients with hepatic type WD showed aggravated symptoms of cirrhosis. The Child‐Pugh grading of three WD patients with hepatic type changed from grade A to B. In addition, liver enzymes, bilirubin, coagulation time, and albumin worsened after drug withdrawal. The occurrence time of neurological symptom aggravation is half a year to a year after drug withdrawal.

Imaging methods were used to analyze the causes of the exacerbation of clinical symptoms in WD patients after drug withdrawal. Previous pathological studies on TX mice (WD animal model) have found that pathological changes in WD brain are different in different disease stages. In the early stage, metal deposition dominated. Demyelination and axonal damage occurred as the disease progresses. At a later stage, the neuron necrosis was obvious. The disease stage of WD should be divided into metal deposition stage, fiber change stage, and neuron necrosis stage according to the characteristics of pathological injury (Zhou et al., 2019). The classification of brain disease stages in WD patients can depend on imaging. SWI is a noninvasive method for determining metal concentration. CP value of SWI of WD is negatively correlated with copper and iron content (Hingwala et al., 2013, Zhou et al., 2019). DTI can indirectly evaluate the integrity of cerebral white matter fibers, and FA value reflects axonal and myelin injury (Hao et al., 2022; Tae et al., 2018). MRS can perform noninvasive detection of brain chemicals, and NAA is an indicator to mark the viability and density of neurons and axons (Igarashi et al., 2021; Whitehead et al., 2022). SWI, DTI, and MRS can be combined to classify the brain stages of WD patients (Zhou et al., 2021). There are obvious abnormalities of CP value on SWI imaging, but no obvious abnormalities of FA value on DTI imaging, and no obvious decrease of NAA peak on MRS imaging, indicating that there is only metal deposition in the brain without obvious fiber damage and neuron necrosis, and this is the stage of metal deposition. When the patient shows obvious abnormal FA value on DTI imaging and no obvious NAA peak drop on MRS imaging, it indicates that there is nerve fiber damage in the brain, but no obvious neuron necrosis, which is classified as the stage of neural fiber damage. When the NAA peak decreases significantly on MRS imaging, the neuron is obviously damaged, which is classified as the stage of neuron necrosis. Most patients with hepatic type WD are in metal deposition stage, patients with nerve fiber injury stage have mild neurological symptoms, and patients with neuron necrosis stage have severe neurological symptoms (Li et al., 2022). It was found in this study that the proportion of WD patients in neuron necrosis stage after drug withdrawal was significantly higher than that before drug withdrawal, suggesting obvious neuron damage occurred in the brain after drug withdrawal. The FA values on DTI imaging of patients decreased after drug withdrawal, suggesting that nerve fiber injury was aggravated. On MRS imaging, NAA/Cr in the subcortical nucleus decreased after drug withdrawal, suggesting that neuronal necrosis was aggravated. The CP value on SWI imaging decreased after drug withdrawal, indicating increased copper deposition in the brain. After drug withdrawal, urine copper also increased significantly, supporting increased copper deposition in the body. Therefore, after drug withdrawal, copper deposition in the brain of WD patients increased and recent nerve damage appeared, which may be the reason for the aggravation of neurological symptoms in patients with drug withdrawal.

In order to observe the response of WD patients to drug therapy, copper excretion treatment was reconducted for 6 months. Since the clinical type of WD patients changed after drug withdrawal, we adjusted the choice of metal chelating agent. Most of the WD patients after drug withdrawal changed to type 2 and 3, so DMPS and DMSA were chosen for these patients. We found that WD patients who resumed metal chelator therapy after drug withdrawal had a poor response to treatment. Neurological symptoms worsened in 34.2% of the patients after treatment, significantly higher than those in the normal treatment group. The neurological symptoms of WD patients aggravated after metal chelating agent therapy is a serious problem. For cerebral type WD patients treated with DPA, the aggravation rate of neurological symptoms was 40%–50%, whereas the aggravation rate of DMPS was about 10%–20% (Cleymaet et al., 2019; Litwin et al., 2017; Schilsky, 2017; Xiao & Fan, 2021). The mechanism of the aggravation of neurological symptoms caused by copper excretion is not completely clear at present, which may be related to the redistribution of copper and nerve injury caused by metal chelating agent. In this study, the proportion of aggravated neurological symptoms of WD patients in drug withdrawal group increased significantly after re‐treatment. The aggravation of neurological symptoms varied with the treatment of different metal chelators. After DPA re‐treatment, neurological symptom scores of WD patients in the withdrawal group were higher than those before treatment. DMPS + DMSA may be recommended for the re‐treatment of WD patients after drug withdrawal.

There are following problems in this study: 1. In this study, 40 WD patients who were reexamined after the withdrawal of metal chelating agent were enrolled, and WD patients with normal treatment were enrolled according to the disease course and clinical classification of these patients. However, most of WD patients who stopped taking drugs had to go back to the clinic again because their symptoms worsened. Patients with symptoms that did not worsen were not included. Therefore, the discontinuation group of patients included in this study cannot fully reflect the situation of all discontinuation patients. Meanwhile, the number of enrolled patients in the whole study was not large, so it was difficult to reflect the overall liver function changes in WD patients. We need to further expand the number of patients and improve the study: 2. In order to study the response of patients to metal chelating agent, this study observed 6 months of re‐treatment. However, the short treatment period of 6 months does not accurately reflect the patient's response to metal chelator therapy. The observation period should be extended further.

In this study, the clinical situation of WD patients with metal chelating agent withdrawal was analyzed, the reasons for drug withdrawal were analyzed, and the prognosis of these patients was tracked. Most of the WD patients who stopped metal chelating agent treatment were with mild neurological symptoms. WD patients who stopped treatment for 6 months to 1 year had aggravated clinical symptoms. Cerebral type WD patients had increased muscle tone, and hepatic type patients had neurological symptoms and deteriorated liver function. After the withdrawal of metal chelating agent, WD patients had increased brain copper deposition and new nerve damage. After the resumption of metal chelating agent therapy, the treatment effect of WD patients was poor. WD patients should undergo long‐term copper excretion treatment and should not easily stop the metal chelating agent.

### PEER REVIEW

The peer review history for this article is available at https://publons.com/publon/10.1002/brb3.3170.

## Data Availability

I confirm that my article contains a Data Availability Statement even if no data is available.
